# A 38‐year‐old female with seizures and a cortico–subcortical mass

**DOI:** 10.1111/bpa.70150

**Published:** 2026-09-20

**Authors:** Marjolein Billen, Annemerel Van Wylick, Anne‐Marie Vanbinst, Hendrik Everaert, Freya Vaeyens, Johnny Duerinck, Bart Neyns, Stefanie Brock

**Affiliations:** ^1^ Department of Pathology Universitair Ziekenhuis Brussel (UZ Brussel) Brussels Belgium; ^2^ Experimental Pathology (EXPA) Vrije Universiteit Brussel (VUB) Brussels Belgium; ^3^ Department of Neurology Universitair Ziekenhuis Brussel (UZ Brussel) Brussels Belgium; ^4^ Department of Medical Oncology Universitair Ziekenhuis Brussel (UZ Brussel) Brussels Belgium; ^5^ Department of Radiology Universitair Ziekenhuis Brussel (UZ Brussel) Brussels Belgium; ^6^ Department of Nuclear Medicine Universitair Ziekenhuis Brussel (UZ Brussel) Brussels Belgium; ^7^ Centre for Medical Genetics, Clinical Sciences, Research group Genetics, Reproduction Development (GRAD) Universitair Ziekenhuis Brussel (UZ Brussel), Vrije Universiteit Brussel (VUB) Brussels Belgium; ^8^ Department of Neurosurgery Universitair Ziekenhuis Brussel (UZ Brussel) Brussels Belgium; ^9^ Laboratory for Medical and Molecular Oncology (LMMO), Translational Oncology Research Center (TORC) Vrije Universiteit Brussel (VUB), Universitair Ziekenhuis Brussel (UZ Brussel) Brussels Belgium

**Keywords:** astrocytoma, glioma, oligoastrocytoma, oligodendroglioma

1

BOX 1 **Virtual glass slide.**

Access at https://isn‐slidearchive.org/?&col=ISN&fol=Archive&file=ID‐7213631.svs.

## 2 CLINICAL HISTORY

A 38‐year‐old woman without significant clinical history experienced two unwitnessed nocturnal episodes over the past 6 months, characterized by tongue biting and urinary incontinence, highly suspicious for tonic–clonic‐generalized seizures. Neurological examination was otherwise unremarkable.

**FIGURE 1:**
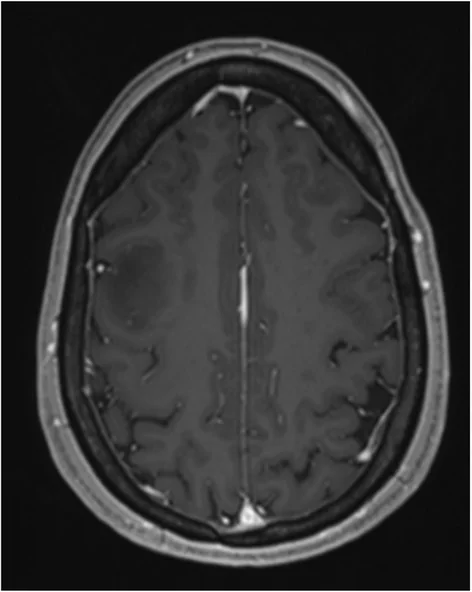
Axial T1‐weighted brain MRI shows a hypointense mass predominantly located in the white matter but focally extending into the precentral gyrus of the right frontal lobe.

MRI of the brain revealed a T2‐ and FLAIR‐hyperintense, T1‐hypointense diffuse mass lesion, located in the right frontal lobe. The lesion was located in the white matter, extending posteriorly into the precentral gyrus. No contrast enhancement, diffusion restriction, or signs of high‐grade biology or transformation were observed. The findings were consistent with a low‐grade glioma (Figure 1). The patient underwent a maximally safe resection.

## 3 FINDINGS

**FIGURE 2:**
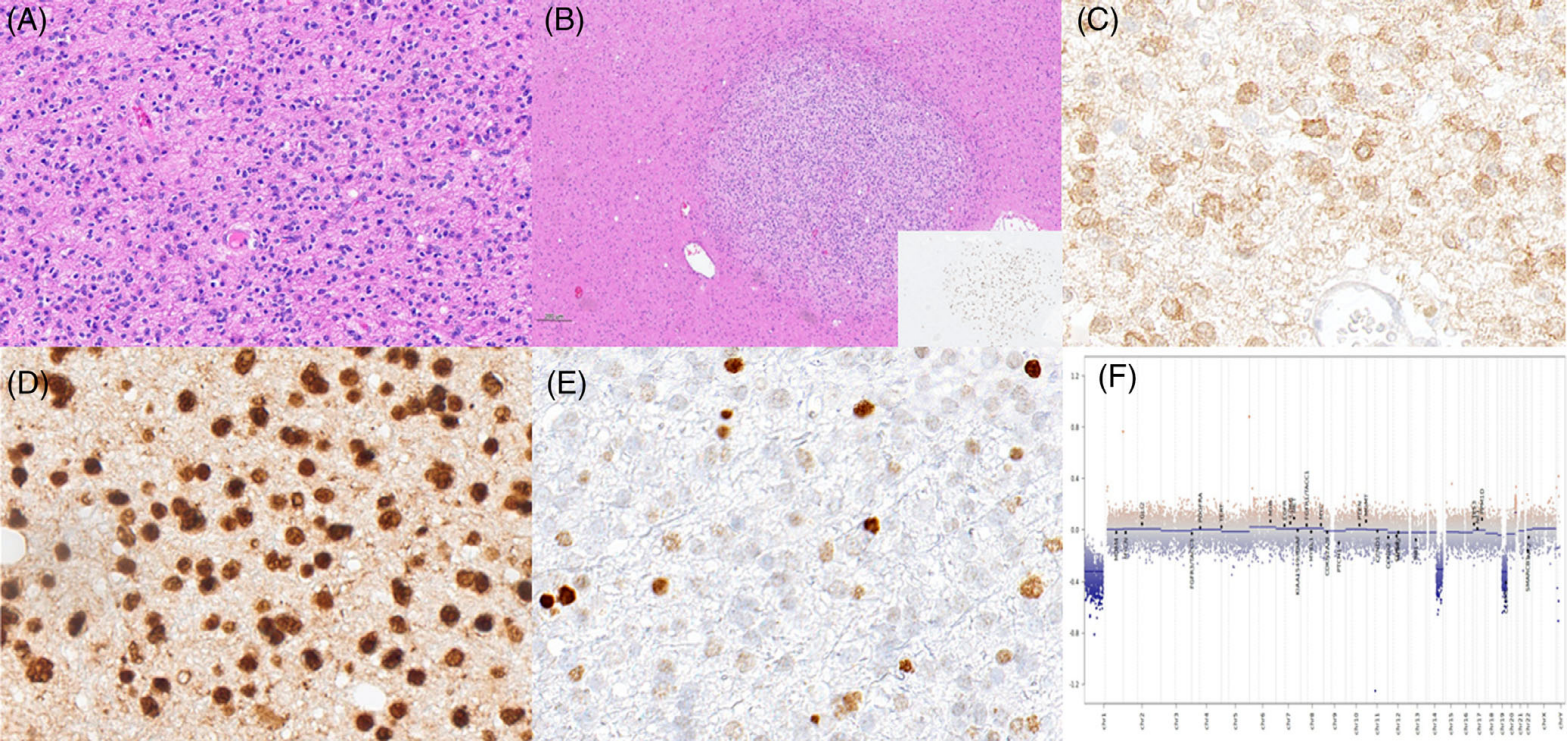
(A) The tumor was moderately cellular and diffusely infiltrating with oligodendrocytic morphology (10×). (B) A small nodule with higher cellularity of predominantly astrocytic morphology (5×) and an increased mitotic index and Ki67 (inset) was observed. (C) Immunohistochemical staining for IDH1, (D) ATRX, and (E) p53 (C–E 40×). (F) Copy number profile derived from methylation array disclosed whole arm 1p/19q deletion.

Histological examination revealed a diffusely infiltrating glial tumor involving the cortex and underlying white matter (Box 1). The tumor was moderately cellular and composed of medium‐sized cells with mildly irregular nuclei (Figure 2A). A substantial proportion of tumor cells exhibited abundant clear cytoplasm with a perinuclear halo, consistent with oligodendroglial morphology, while intermingled smaller cells showed a morphology reminiscent of astrocytic lineage. The background was prominently fibrillary. No necrosis or microvascular proliferation was observed, and the vasculature lacked a characteristic “chicken‐wire” pattern. The mitotic count was low (1 mitosis/10 high power fields (HPF), 2 mitoses/2.6mm^2^) and proliferation index (Ki67) in the tumor ranged between 5% and 8%. Very focally, a proliferative nodule was identified, showing increased mitotic activity of up to 14 mitoses per 10 HPF and an increased Ki67 index of approximately 20% (Figure 2B).

Immunohistochemical analysis demonstrated strong positivity for GFAP and partial positivity for OLIG2 (>60% of tumor cells). IDH1 R132H immunostaining was positive (Figure 2C). ATRX expression was retained, and p53 was positive in approximately 5% of tumor cells (Figure 2D,E). H3K27me3 expression was retained. Tumor cells were negative for Vimentin.

DNA based next‐generation sequencing revealed an *IDH1* R132H pathogenic variant with coexisting pathogenic variants in *TERT* promoter and *ATRX*. Notably, the allele frequency of the *IDH1*, *TERT*, and *ATRX* pathogenic variants was similar, being 33%, 32% and 36%, respectively. A subclonal pathogenic variant in *TP53* was identified at 5% allele frequency. Fluorescence in situ hybridization demonstrated 1p/19q co‐deletion, with 72% and 75% of analyzed cells showing loss of 1p36 and 19p13, respectively. No homozygous or heterozygous *CDKN2A/B* deletion was detected.

DNA methylation profiling (Heidelberg Brain Tumor Classifier v12.8) classified the tumor as a “diffuse glioma, IDH‐mutant” with a high confidence score (0.99), with a subclassification suggestive of “oligodendroglioma, IDH‐mutant and 1p/19q‐codeleted” (score 0.65), and a lower score for “astrocytoma, IDH‐mutant, lower grade” (score 0.33). Copy number variation analysis confirmed 1p/19q co‐deletion (Figure 2F).

## 4 DIAGNOSIS

Dual‐genotype IDH‐mutant glioma, consistent with an oligoastrocytoma, not elsewhere classified (NEC), WHO CNS 2021 predominantly Grade 2, but with a very focal area of increased mitotic activity suspicious for evolution toward Grade 3.

## 5 DISCUSSION

Dual‐genotype oligoastrocytomas are rare and currently not recognized as a distinct entity by the WHO CNS 2021 classification and cIMPACT‐NOW recommendations [1]. Although morphological overlap between IDH‐mutant oligodendrogliomas and astrocytomas is recognized, distinction between these two entities is now primarily based on molecular features. The diagnosis of oligodendroglioma, IDH‐mutant in our case was supported by pathogenic variants in *IDH1* R132H and *TERT*, and 1p/19q co‐deletion. However, the presence of concurrent pathogenic variants in *IDH1*, *ATRX*, and *TP53* is considered mutually exclusive with 1p/19q‐codeleted oligodendrogliomas, and instead favor a diagnosis of astrocytoma, IDH‐mutant [1]. The coexistence of these overlapping molecular features led to a diagnosis of a dual‐genotype oligoastrocytoma. Interestingly, *ATRX* was diffusely retained in tumor cells in our patient despite the detection of a pathogenic variant. This variant induced a premature stop codon located at the C‐terminal end of the protein, potentially explaining maintained aberrant protein expression.

Previous studies of morphologically defined oligoastrocytomas have reported spatially distinct or diffusely intermixed oligodendroglial and astrocytic populations. However, immunohistochemical and molecular profiles did not overlap, segregating into 1p19q‐co‐deleted, ATRX retained oligodendroglial, and 1p19q‐intact, *ATRX* and *TP53* mutant astrocytic populations [2, 3]. In the present case, immunohistochemically no separate populations were present, variant allele frequencies were similar for *IDH1*, *ATRX*, and *TERT*, and 1p19q co‐deletion was present in the majority of cells. Although the subclonal *TP53* variant may originate from the intermixed astrocytic component and/or the proliferative nodule, this could not be confirmed by immunohistochemistry. Given that molecular profiling was performed on DNA extracted from the whole tissue section, it could not be established whether the identified molecular alterations were restricted to specific histological components or shared across them.

The absence of necrosis and microvascular proliferation supports WHO CNS Grade 2; however, the presence of a focal area with increased mitotic activity and an elevated proliferative index raised concern for progression toward WHO CNS Grade 3. While this has also been acknowledged by c‐IMPACT‐NOW update 12, which suggests a cut‐off of ≥6 mitoses/10 HPF of 0.24 mm^2^ in oligodendrogliomas, lack of contrast enhancement on MRI, as in our patient, appears to be associated with prolonged survival [1].

In conclusion, this case highlights how detailed molecular studies, including NGS and methylation profiling, may disclose additional unexpected findings in IDH‐mutant gliomas, with an otherwise typical morphological and immunohistochemical profile.

## AUTHOR CONTRIBUTIONS

MB: data collection and manuscript drafting. AV, HE, FV, AW, JD, BN: data collection, review of the manuscript. SB: conceptualization, data collection, manuscript drafting.

## CONFLICT OF INTEREST STATEMENT

The authors declare no conflicts of interest.

## ETHICS STATEMENT

All data related to this case are deidentified. Ethics commission approval for this study was granted by the ethics committee of the Universitair Ziekenhuis Brussel.

## Data Availability

The data that support the findings of this study are available from the corresponding author upon reasonable request.
